# *Fgf10^+^* progenitors give rise to the chick hypothalamus by rostral and caudal growth and differentiation

**DOI:** 10.1242/dev.153379

**Published:** 2017-09-15

**Authors:** Travis Fu, Matthew Towers, Marysia A. Placzek

**Affiliations:** The Bateson Centre and Department of Biomedical Science, University of Sheffield, Sheffield S10 2TN, UK

**Keywords:** Fgf10, Shh, Chick, Hypothalamus, Prechordal mesendoderm

## Abstract

Classical descriptions of the hypothalamus divide it into three rostro-caudal domains but little is known about their embryonic origins. To investigate this, we performed targeted fate-mapping, molecular characterisation and cell cycle analyses in the embryonic chick. Presumptive hypothalamic cells derive from the rostral diencephalic ventral midline, lie above the prechordal mesendoderm and express *Fgf10*. *Fgf10^+^* progenitors undergo anisotropic growth: those displaced rostrally differentiate into anterior cells, then those displaced caudally differentiate into mammillary cells. A stable population of *Fgf10^+^* progenitors is retained within the tuberal domain; a subset of these gives rise to the tuberal infundibulum – the precursor of the posterior pituitary. Pharmacological approaches reveal that Shh signalling promotes the growth and differentiation of anterior progenitors, and also orchestrates the development of the infundibulum and Rathke's pouch – the precursor of the anterior pituitary. Together, our studies identify a hypothalamic progenitor population defined by *Fgf10* and highlight a role for Shh signalling in the integrated development of the hypothalamus and pituitary.

## Abstract (precis)

**Summary:** Anterior and then mammillary hypothalamic cells differentiate from *Fgf10*^+^ progenitors that are retained as central tuberal hypothalamic cells. Shh is required for anterior regionalisation and also for integrated hypothalamic/pituitary development.

## INTRODUCTION

The hypothalamus is an evolutionarily ancient part of the brain that acts as a master homeostatic regulator. It controls the circadian cycle, endocrine function, energy and stress balance through the concerted activity of its resident neurons. These neurons are located in nuclei that are assigned to distinct rostro-caudal domains: anterior, tuberal and mammillary (Swanson, 1987). Particular subclasses of hypothalamic neurons, termed neuroendocrine neurons, project axons to the median eminence and posterior pituitary – the ventral-most regions of the tuberal hypothalamus. Here, they release neurohormones that act directly, or indirectly, via cells of the adjacent anterior pituitary, to govern endocrine function (Swanson, 1987). An understanding of hypothalamic development is therefore fundamental to our understanding of neuroendocrine axis formation and function in health and disease.

In the embryonic chick, as in all vertebrates examined, conserved molecular features define the developing hypothalamus and demarcate its antero-posterior (future rostro-caudal) domains. At neural plate stages, presumptive hypothalamic and telencephalic territories can be distinguished through homeodomain (HD) protein expression profiles: the presumptive telencephalon co-expresses *Foxg1* and *Six3*, whereas the presumptive hypothalamus expresses only *Six3* (Bovolenta et al., 1998; Bell et al., 2001; Chapman et al., 2002; Ahlgren et al., 2003; Sanchez-Arrones et al., 2012). At HH17, after neural tube formation, *Shh*, *Fgf8*, *Fgf10*, *Fgf19* and *Emx2* become detected in apparently non-overlapping progenitor domains along the antero-posterior axis of the developing hypothalamus (Schubert and Lumsden, 2005; García-Calero et al., 2006; Hurtado and Mikawa, 2006; Manning et al., 2006; Gimeno and Martinez, 2007; Parkinson et al., 2010). Previous studies have begun to reveal how these domains develop. In the anterior neural plate/neural tube, rostral diencephalic ventral midline (RDVM) cells are specified through signals that derive from underlying prechordal mesendoderm (PM) (Dale et al., 1997, 1999; reviewed by Placzek and Briscoe, 2005). In a subset of RDVM cells, *Shh* is then downregulated by BMPs that derive from the PM. Upregulation of BMP signalling in RDVM cells leads to expression of *Tbx2*, a step that promotes the development of RDVM cells to *Fgf10^+^* and *Emx2^+^* progenitors (Manning et al., 2006). Lineage-tracing studies show that *Fgf10^+^* progenitors give rise to the infundibulum, an outgrowth of the tuberal hypothalamus (Pearson et al., 2011), whereas *Emx2* appears to mark mammillary progenitors (Manning et al., 2006). As *Shh* is downregulated in RDVM cells, however, it becomes detected in adjacent anterior progenitors (Ohyama et al., 2005; Manning et al., 2006; Aglyamova and Agarwala, 2007).

Currently, the origins of anterior *Shh^+^*, tuberal *Fgf10^+^* and mammillary *Emx2^+^* progenitors, and their relationship with each other and with differentiated cells in the adult hypothalamus remain unresolved. Fate-mapping studies in the chick have provided inconsistent conclusions (Garcia-Lopez et al., 2004; Manning et al., 2006; Pearson et al., 2011) and lineage-tracing studies in the mouse have relied on promoters/enhancers that are not specific to hypothalamic progenitors (Alvarez-Bolado et al., 2012): as yet, no single early marker has been identified that delineates or defines the hypothalamic progenitor state. The controversy surrounding the position of hypothalamic progenitors fuels debate into which surrounding tissues may influence its development. Thus, although many studies highlight the importance of the PM (Shimamura and Rubenstein, 1997; Dale et al., 1997; Ohyama et al., 2005; Patten et al., 2003; Pera and Kessel, 1997; reviewed by Bedont et al., 2015; Burbridge et al., 2016), other studies suggest the PM is distant and thus less significant (Garcia-Calero et al., 2008; reviewed by Puelles and Rubenstein, 2015).

Here, we perform studies in the embryonic chick to address questions about the origin of the hypothalamus and the emergence of distinct rostro-caudal progenitor domains. We create an extensive fate-map of cells in the ventral midline/adjacent basal plate of the prosencephalon to show that hypothalamic progenitors lie above the PM at 9-10 somites. We reveal that these progenitors express *Fgf10* and give rise to anterior and mammillary progenitor domains by bidirectional anisotropic growth and differentiation. Following this, the remaining Fgf10-positive progenitors give rise to cells within the tuberal domain. Finally, we uncover a crucial role for Shh signalling, both in growth and differentiation of anterior progenitors and in pituitary development (see Carreno et al., 2017). Together, our studies reveal how spatially distinct rostro-caudal domains of the hypothalamus are established.

## RESULTS

### Characterising the embryonic chick hypothalamus

**Fig. 1.**
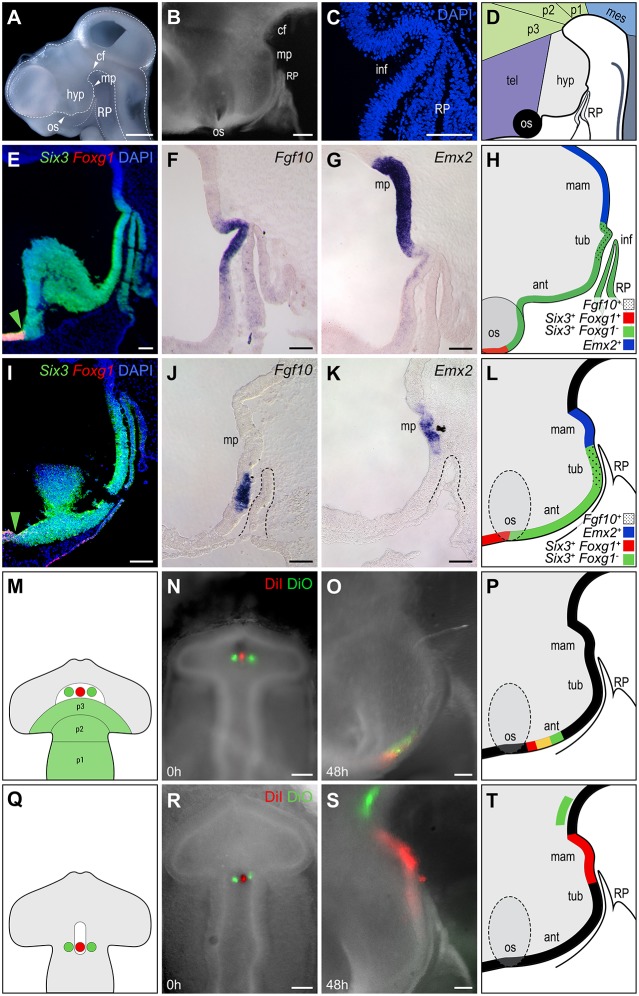
**Fate-mapping defines the extent of hypothalamic progenitors.** (A-D) The position of the hypothalamus relative to adjacent structures, including Rathke's pouch (RP), shown: (A) in E5 whole-mount side view (dashed lines outline the neuroepithelium and RP); (B) after hemi-dissection at E3; (C) after sagittal sectioning (a high-power view of E5 chick to show the proximity of the infundibulum and RP); and (D) schematically (E5 chick). (E-L) Sagittal sections at E5 (E-H) or E3 (I-L) analysed by double fluorescent *in situ* hybridisation for *Six3*/*Foxg1* (E,I) or by single *in situ* hybridisation for *Fgf10* (F,J) or *Emx2* (G,K), and illustrated schematically in H,L. Dashed lines in J,K outline RP. Green arrowheads in E,I indicate the telencephalic-hypothalamic boundary. (M-T) Fate-mapping of the medial prosencephalon (M-P) or prosencephalic neck (Q-T). (M,Q) Schematic representation of prosencephalon. White areas indicate the suggested position of hypothalamic progenitors from Garcia-Lopez et al. (2004) (M) or Manning et al. (2006) and Pearson et al. (2011) (Q). Circles show the position of focal DiI (pink) and DiO (green) injections. (N) Dorsal view of a 10-somite embryo after triple injection of DiI in the ventral midline of the medial prosencephalon and of DiO in adjacent basal plate (*n*=2). (O,P) Hemi-dissected side view of the same embryo incubated to HH20 (O), schematically represented in P. Labelled cells lie posterior to the optic stalk. (R) Dorsal view of a 9-somite embryo after triple injection of DiI in the ventral midline of the prosencephalic neck and of DiO in adjacent basal plate (*n*=2). (S,T) Hemi-dissected side view of the same embryo incubated to HH20 (S), schematically represented in T. DiI-labelled cells populate the ventral hypothalamus, extending from the mammillary pouch to the tuberal hypothalamus. DiO-labelled cells populate the basal plate of p2. Scale bars: 1 mm in A; 200 µm in B; 100 µm in C-S. ant, anterior hypothalamus; cf, cephalic flexure; hyp, hypothalamus; mam, mammillary hypothalamus; mes, mesencephalon; mp, mammillary pouch; os, optic stalk; p1-3, prosomeres 1-3; RP, Rathke's pouch; tel, telencephalon; tub, tuberal hypothalamus.

At embryonic days (E)3 (HH18-20) to E5 (HH26), the chick hypothalamus is clearly visible as a ventral protrusion with a rostral limit at the optic stalk and a caudal limit at the mammillary pouch (Fig. 1A; Fig. S1). A novel hemi-dissection technique preserves the association of Rathke's pouch (RP) with the hypothalamus (Fig. 1B). At E5, the tip of RP underlies the future infundibulum, a ventral outgrowth of the tuberal hypothalamus (Fig. 1C,D; Pearson et al., 2011). *In situ* hybridisation and analyses of sagittal sections at E5 reveals the telencephalic (*Foxg1^+^Six3^+^*)*-*hypothalamic (*Foxg1^−^Six3^+^*) boundary and shows that *Six3* extends to the infundibulum, i.e. defines anterior and tuberal progenitor domains (Fig. 1E). Tuberal *Fgf10^+^* progenitors lie above RP (Fig. 1F) and anterior to *Emx2^+^* mammillary progenitors (Fig. 1G). Thus, at E5, anterior, tuberal and mammillary progenitor domains can be defined on the basis of gene expression profiles against known morphological landmarks: *Six3^+^Foxg1^−^Fgf10^−^* anterior progenitors extend from the optic stalk to RP; *Six3^+^Foxg1^−^Fgf10^+^* tuberal progenitors lie above RP, and *Emx2^+^Six3^−^Fgf10^−^* mammillary progenitors extend from the infundibulum into the mammillary pouch (Fig. 1H).

Anterior, tuberal and mammillary progenitor domains can similarly be identified at E3. *Six3* is expressed in the *Foxg1^+^* telencephalon, and in anterior and tuberal progenitors: its caudal limit aligns with the tip of RP (Fig. 1I). *Fgf10* is expressed in tuberal progenitors that overlie RP (Fig. 1J) and *Emx2* is expressed in mammillary progenitors (Fig. 1K). Thus, the E3 hypothalamus can be subdivided into a *Foxg1^−^Six3^+^Fgf10^−^* anterior progenitor domain rostral to RP, a *Six3^+^Fgf10^+^Emx2^−^* tuberal progenitor domain, which overlies RP, and a *Emx2^+^Six3^−^Fgf10^−^* mammillary progenitor domain (Fig. 1L). Furthermore, anterior, tuberal and mammillary progenitor domains can be conclusively identified in hemi-dissected E3 embryos, based on their position relative to RP.

We next determined the extent of hypothalamic progenitors in the ventral midline of 9-10 somite embryos. Previous studies have suggested very different origins for hypothalamic cells: chimaeric interspecies grafting studies have suggested that hypothalamic progenitors occupy the middle of the prosencephalon, anterior to prosomere 3 (Fig. 1M, white region; Garcia-Lopez et al., 2004), whereas focal-labelling studies have placed hypothalamic progenitors more posteriorly, at the prosencephalic neck (Fig. 1Q, white region; Manning et al., 2006; Pearson et al., 2011). We therefore targeted the extremes of each region (Fig. 1M,Q), injecting DiI (red) into the ventral midline and DiO (green) into adjacent basal plate cells, then analysing the position of descendants at E3. Ventral midline (DiI^+^) and basal plate cells (DiO^+^) in the middle of the prosencephalon contributed exclusively to the rostral-most part of the anterior hypothalamus, as determined in hemi-dissected view relative to the optic stalk and RP, and showed little growth (Fig. 1N-P). Transverse sections revealed that ventral midline and basal plate cells did not mix, but maintained their relative mediolateral position (Fig. S2). By contrast, ventral midline (DiI^+^) cells at the prosencephalic neck showed extensive expansion along the rostro-caudal axis and gave rise to mammillary and caudo-tuberal progenitor domains, as determined in hemi-dissected view relative to RP (Fig. 1R-T). Basal plate progenitors (DiO^+^) at the prosencephalic neck did not contribute to the hypothalamus but instead appeared to contribute to prosomere 2 (Fig. 1R-T). These results show that at 9-10 somites, hypothalamic progenitors extend from the prosencephalic neck to the middle of the prosencephalon. Their descendants populate the entire rostro-caudal extent of the hypothalamus, from the optic stalk to the mammillary pouch.

### Hypothalamic progenitor domains originate in the RDVM above the PM

**Fig. 2.**
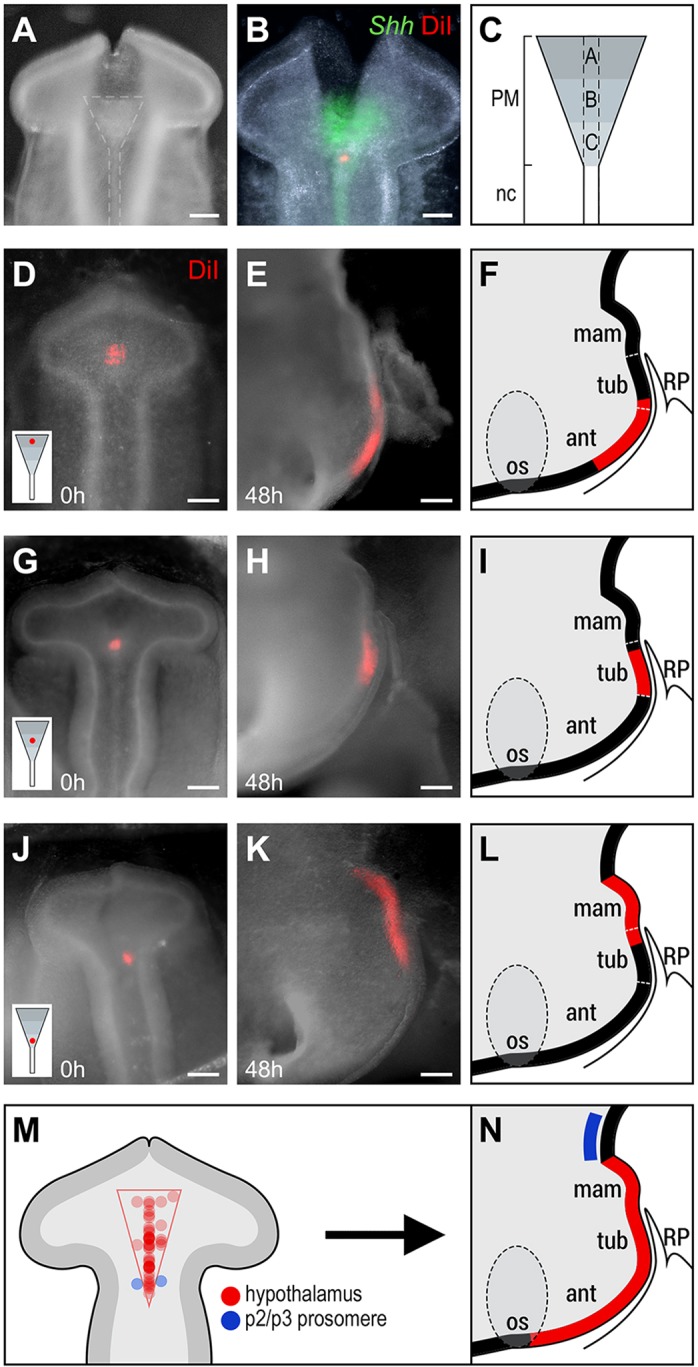
**At 9-10 somites, hypothalamic progenitors lie above the PM.** (A-C) Targeting neuroepithelium above the PM. (A) Whole-mount dorsal view of a 10-somite embryo after incision of the dorsal neural tube reveals the PM and notochord (dashed lines). (B) Double analysis of DiI and *Shh*. (C) Schematic showing subdivision of the PM/RDVM into three regions: A (anterior), B (middle) and C (posterior). Dashed lines indicate position of the ventral midline. (D-L) Representative chicks, showing the fate of neuroepithelial cells after focal DiI injections to regions A (*n*=4; D-F), B (*n*=4; G-I) and C (*n*=2; J-L). (D,G,J) Whole-mount dorsal view of 9-10 somite embryos showing DiI injection in regions A (D), B, (G) and C (J). Inset shows injection site relative to the PM. (E,F,H,I,K,L) Whole-mount side views (E,H,K) after development to HH20 and hemi-dissection to show position of DiI-labelled cells, schematically represented in F,I,L. (M,N) Summary. Schematics of 9-10 somite prosencephalon, showing injection sites of all fate-mapping experiments relative to the prosencephalic neck and PM (M; *n*=43 injections), and position of descendants at E3 (N). Circle intensity in M indicates multiple separate injections. Scale bars: 100 µm. ant, anterior hypothalamus; mam, mammillary hypothalamus; os, optic stalk; RP, Rathke's pouch; tub, tuberal hypothalamus.

Many studies suggest that the PM induces the hypothalamus and then directs its subsequent development (reviewed by Bedont et al., 2015; Burbridge et al., 2016), but its exact position relative to hypothalamic progenitors remains unclear. Intriguingly, we noted that progenitors that give rise to the rostral-most anterior hypothalamus (Fig. 1M-P) appear to lie close to the anterior-most PM, the outline of which is apparent under bright-field view (Fig. 2A). We therefore asked whether progenitors that fate-map to the hypothalamus lie over the PM in the 9-10 somite embryo. To show that we could accurately target RDVM cells, we labelled them with DiI and immediately analysed embryos for *Shh*: DiI-labelled cells were observed above the *Shh^+^* PM (Fig. 2B; and data not shown). To fully explore the extent to which PM cells underlie hypothalamic progenitors, DiI was targeted to the ventral midline overlying anterior (A), medial (B) and posterior (C) regions of the PM (Fig. 2C). Embryos were developed until E3 or E5, analysed in hemi-dissected view (Fig. 2D-L), then sectioned and analysed for marker expression to distinguish anterior, tuberal and mammillary progenitor domains (Figs S3 and S4).

Targeting relative to the PM produced consistent fate-maps. Neuroepithelial cells above the anterior PM (region A) gave rise to anterior and tuberal progenitors (*n*=4/4; Fig. 2D-F). Transverse sectioning, and labelling of alternate sections for *Fgf10*, confirmed that DiI was detected in the *Fgf10^−^* anterior progenitor domain and in the *Fgf10^+^* rostro-tuberal progenitor domain (Fig. S3)*.* No labelled cells were detected in the mammillary progenitor domain (not shown). Neuroepithelial cells above region B gave rise to tuberal progenitors (*n*=4/4; Fig. 2G-I), confirmed after transverse sectioning and labelling of alternate sections for *Fgf10* (Fig. S3). Neuroepithelial cells above the posterior PM (region C) gave rise to *Emx2^−^* caudo-tuberal and *Emx2^+^* mammillary progenitors (*n*=4/4; Fig. 2J-L; Fig. S4). Triple fate-mapping showed that, despite extensive growth along the rostro-caudal axis, cells maintain their relative position and show some growth/migration along the medio-lateral axis (Fig. S5A-I).

To begin to define the position of hypothalamic progenitors along the medio-lateral axis, we targeted ventral midline cells and adjacent basal plate cells (DiO) in regions overlying the PM. Basal plate progenitors contributed to basal hypothalamic regions. They showed growth along the rostro-caudal axis, but to a lesser extent than ventral midline progenitors (Fig. S5J-N). Together, these studies define the extent of hypothalamic progenitors in the ventral midline in the 9-10 somite embryo and begin to define their position in the medio-lateral axis. The position and shape of the PM suggests that it underlies and defines the limit of RDVM progenitors which will populate the forming anterior, tuberal and mammillary hypothalamus at E3 (Fig. 2M,N). Over this time, these progenitors expand in number; they grow extensively along the rostro-caudal axis, but maintain their relative position. Together, our studies show that hypothalamic progenitors become regionalised according to their position along the rostro-caudal axis, but that progenitors targeted above any region of the PM contribute some descendants to the tuberal progenitor domain.

### Anterior and mammillary progenitors derive from *Fgf10^+^* hypothalamic progenitors

We next analysed *Six3*, *Fgf10* and *Emx2* at 9-10 somites to address whether, at the time of fate-mapping, hypothalamic progenitors express these markers, and, if so, whether distinct progenitor domains are already apparent.

**Fig. 3.**
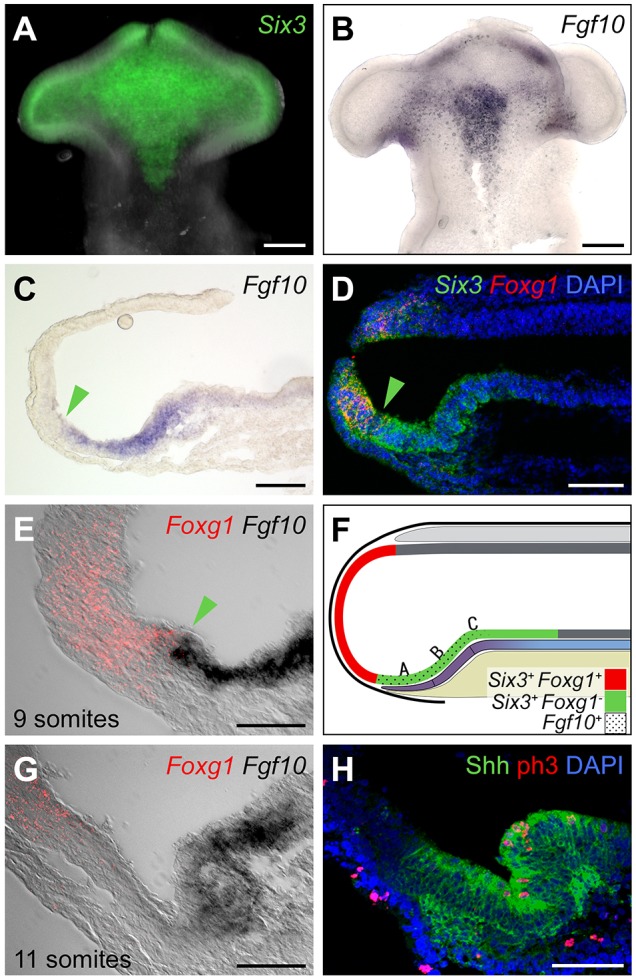
**Hypothalamic progenitors at 9-10 somites express *Fgf10.*** (A,B) Ventral flat-mount views of 10-somite isolated neuroepithelium after *in situ* hybridisation for *Six3* (A; *n*=4) or *Fgf10* (B; *n*=6). (C-F) Sagittal sections of 9-10 somite embryos. (C) *In situ* hybridisation for *Fgf10* (*n*=5). (D-F) Double *in situ* hybridisation to detect *Six3/Foxg1* (D) or *Foxg1*/*Fgf10* (E) (*n*=3 each), shown schematically in F. *Fgf10^+^* cells lie above the PM (purple indicates prechordal mesoderm; beige indicates endoderm) and abut the *Foxg1^−^* telencephalon. A,B,C refer to fate-mapped regions. Green arrowheads in C-E show the position of the tel-hyp boundary. (G,H) Sagittal sections of 11-somite embryos. (G) *In situ* hybridisation for *Foxg1*/*Fgf10*: anterior *Fgf10^−^* cells are apparent. (H) Immunohistochemical analysis for Shh/phospho-H3. A significantly higher density of phospho-H3^+^ cells is detected in tuberal compared with anterior domains (9.13±0.74 versus 3.13±0.72 phosphoH3^+^ cells/4.5×10^−6^ mm^3^; *P*<0.0001; *n*=8 sections; 4 embryos). Scale bars: 100 µm in A-D; 50 µm in E,G,H.

Whole-mount *in situ* hybridisation shows that *Six3* is expressed throughout the ventral prosencephalon, including the telencephalon and hypothalamus. Expression is wide anteriorly and progressively narrower posteriorly (*n*=4; Fig. 3A). *Fgf10* is similarly detected ventrally, but is confined to the middle and posterior prosencephalon (*n*=6; Fig. 3B), where its profile is similar to that of *Six3* (i.e. tapering posteriorly). Expression of *Emx2* is undetectable (Manning et al., 2006). Comparison with our fate-mapping data thus suggests that, in the 9-10 somite chick, hypothalamic progenitors express *Six3* and *Fgf10*, but not *Emx2*.

Analysis of *Fgf10* in sagittal view suggests that *Fgf10^+^* progenitors may extend as far as the telencephalic boundary (Fig. 3C). To confirm this, we performed double labelling of *Six3*/*Foxg1* or *Fgf10*/*Foxg1*, followed by sagittal sectioning. This reveals that, at 9-10 somites, the anterior limit of *Fgf10* immediately abuts the telencephalic boundary marked by *Foxg1* (Fig. 3D-F). Therefore, at the time of fate-mapping, progenitors characteristic of tuberal (*Six3^+^Fgf10^+^*), but not anterior (*Six3^+^Fgf10^−^*) or mammillary (*Fgf10^−^Emx2^+^*), domains are present. Anterior progenitors can be detected only at 11 somites, approximately 1.5-2 h later than the fate-mapping studies (Fig. 3G), and appear concomitant with a characteristic folding of the hypothalamic floor and a distancing of the PM and the telencephalic/hypothalamic boundary (Fig. 3G,H). Mammillary progenitors appear still later, and are first detected at HH17 (Fig. S6). Together with our fate-mapping analysis, these results show that at 9-10 somites, the presumptive hypothalamus overlies the PM and is composed of *Fgf10^+^* progenitors that give rise to anterior, then mammillary and tuberal domains.

### Anterior and then mammillary domains arise by anisotropic growth

**Fig. 4.**
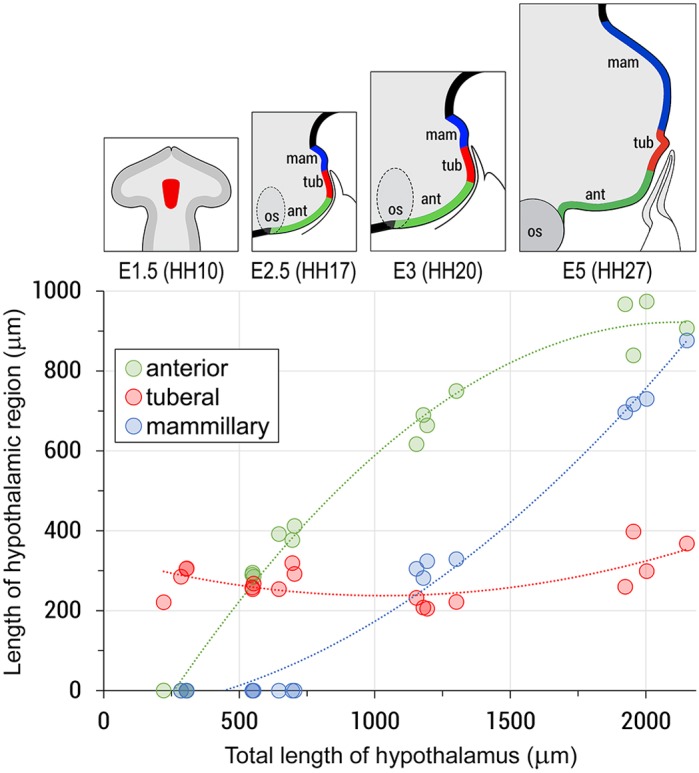
**Growth of the hypothalamus is anisotropic.** Scatter plot showing growth of anterior (green), tuberal (red) and mammillary (blue) progenitors from HH10 (E1.5) to HH27 (E5). Anterior and posterior domains emerge and grow by 922±63 µm and 910±85 µm; *n*=4 each; the lengths of the tuberal domains do not alter significantly (279±40 to 256±18, *P*>0.05; *n*=4 each). Circles represent analyses of individual chicks. Lines of best fit were generated using Microsoft Excel. Top schematics illustrate the growth of anterior (green) and posterior (blue) progenitors, and the relatively unchanged length of *Fgf10^+^* hypothalamic (prospective tuberal) progenitors (red).

To further understand how anterior, mammillary and tuberal domains of the hypothalamus develop from *Fgf10^+^* progenitors, we measured the rostro-caudal length of all three regions at HH10, HH15, HH17, HH20 and HH27 (Fig. 4). Despite the entire hypothalamic region expanding in size (∼8-fold expansion) over this period, the *Fgf10^+^* progenitor domain undergoes remarkably little change in growth and remains a constant size (Fig. 4, red circles). By contrast, the anterior progenitor domain appears and increases dramatically in size between HH10^+^ and HH20 (Fig. 4, green circles). As growth of the anterior progenitor domain subsides, the mammillary progenitor domain forms and shows substantial growth from HH18 to HH27 (Fig. 4, blue circles; see also Fig. 1G,K). *Fgf10^+^* progenitors are retained centrally and contribute exclusively to the tuberal domain. These data reveal the dynamic and bi-directional growth of the anterior and mammillary hypothalamus from *Fgf10^+^* progenitors. A defining feature of the tuberal domain, the infundibulum, emerges over E4-E7 (Pearson et al., 2011), i.e. after the major growth phase of the anterior progenitor domain.

### Anterior and mammillary progenitor domains arise from highly proliferative *Fgf10^+^* progenitors

**Fig. 5.**
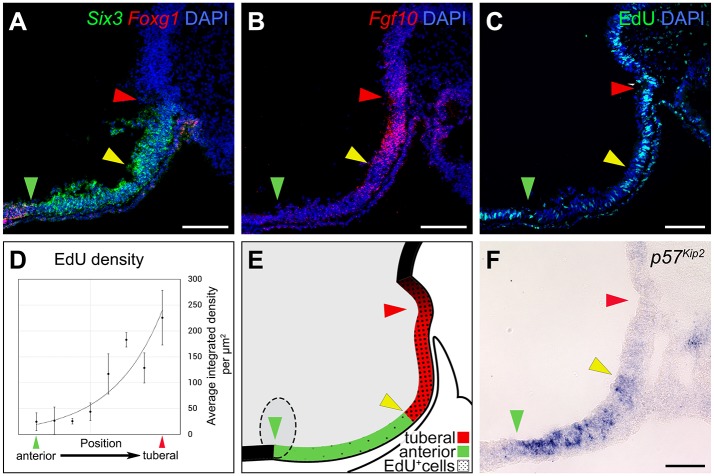
**Anterior progenitors grow and differentiate from *Fgf10*^+^ hypothalamic progenitors.** (A-C) Serial adjacent sagittal sections of HH15 embryos (*n*=3), analysed for *Six3/Foxg1* (A), *Fgf10* (B) or EdU (C). Green arrowheads show the tel-hyp boundary; the region between the yellow and red arrowhead indicates the *Six3^+^ Fgf10^+^* progenitor domain. (D,E) Quantitative analysis of EdU labelling through anterior-tuberal progenitor domains (D; *n*=4), depicted schematically in E (arrowheads as in A-C). (F) Sagittal section of a HH15 embryo, analysed for *p57^kip2^* expression (*n*=4). Expression is confined to anterior progenitors, and is higher rostrally (green arrowhead) and lower caudally (yellow arrowhead). Scale bars: 100 µm.

We next addressed whether localised proliferation could contribute to bi-directional growth of anterior and mammillary progenitor domains. Previous studies have shown that FGF signalling governs proliferation of hypothalamic progenitors (Pearson et al., 2011), implying that *Fgf10^+^* progenitors self-propagate and are responsive to FGFs acting as autocrine signals. However, it remains possible that once initial pools of *Fgf10^−^* anterior and mammillary progenitors have formed, they then expand in response to FGFs acting as paracrine signals from presumptive tuberal regions. To distinguish these possibilities, we first analysed expression of *Pea3*, a marker of FGF-responding cells. From HH9 to HH26, *Pea3* is expressed in *Fgf10^+^* progenitors, but not in adjacent anterior and mammillary progenitors, suggesting that FGF signalling is autocrine (Fig. S7; data not shown). Next, we performed acute EdU labelling assays at HH15 and HH20, times when, respectively, anterior and mammillary progenitor domains expand rapidly. At HH15, labelling of serial adjacent sagittal sections shows that the *Foxg1^−^Six3^+^Fgf10^−^* anterior progenitor domain (Fig. 5A-C, green to yellow arrowheads) contains a lower number of EdU-labelled cells compared with the *Foxg1^−^Six3^+^Fgf10^+^* tuberal progenitor domain (Fig. 5A-C, yellow to red arrowheads). Quantification of EdU fluorescence measured as density through the anterior/tuberal domains (Fig. 5C, green to red arrowheads) reveals a gradient of proliferation: EdU^+^ progenitors are detected at high density in the tuberal domain and at diminishing density through the anterior domain (Fig. 5D,E). Similarly, at HH20, the *Emx2^+^* domain contains a lower density of EdU-labelled cells compared with the tuberal domain (Fig. S8). These results, together with our fate-maps, suggest that anterior and mammillary progenitor domains develop from highly proliferating *Fgf10^+^* progenitors.

The decline in proliferation towards the rostral and caudal ends of both anterior and mammillary progenitor domains implies that cells begin to differentiate once displaced from the *Fgf10^+^* domain. To explore a mechanism for this, we analysed expression of Cip/Kip family members, which encode cell cycle regulators of the G1-to-S-phase transition that promote cell cycle exit and differentiation (Galderisi et al., 2003). We detected *p57^Kip2^*, but not *p27^Kip1^* or *p21^Cip1^* (Fig. 5F, Fig. S9; and data not shown). At HH15, *p57^Kip2^* is detected only in the anterior progenitor domain. Expression is graded, with highest levels detected in rostral-most and lowest levels in caudal-most anterior progenitors (Fig. 5F, green and yellow arrowheads, respectively). At HH18, *p57^Kip2^* is also detected in caudal mammillary progenitors, but is, at best, only weakly detected in tuberal progenitors (Fig. S9A-C). By HH26, *p57^Kip2^* shows graded expression through the mammillary domain, with highest levels detected most caudally (Fig. S9D-I). Thus, opposing gradients of proliferation and differentiation underlie the bi-directional growth of the developing anterior and mammillary hypothalamus.

### Upregulation of *Shh* and *p57^Kip2^* in anterior progenitors

**Fig. 6.**
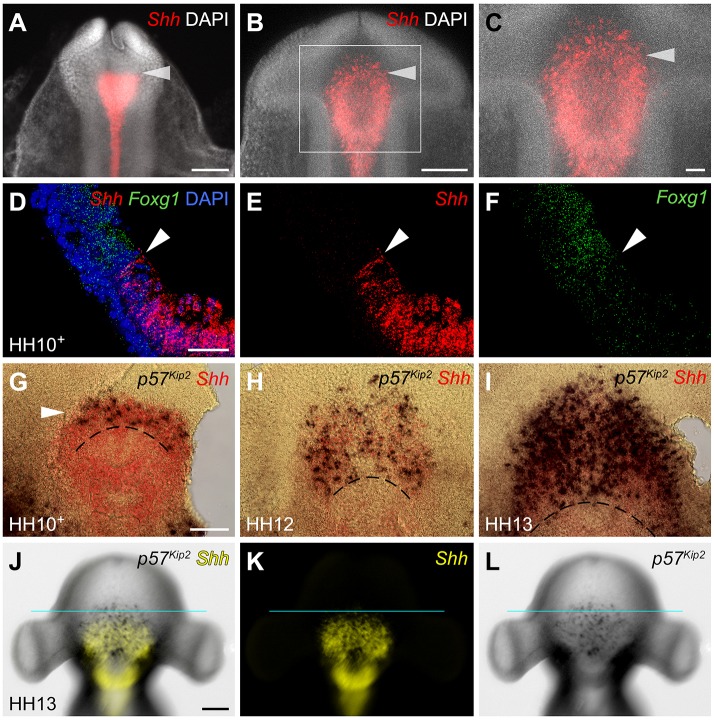
**Onset of *Shh* and *p57^Kip2^* characterises anterior hypothalamic cells.** (A-C) Ventral view of flat-mounted embryos at 8 (A) or 11 (B,C) somites after *in situ* hybridisation to detect *Shh* (*n*=5 each). Arrowhead indicates the position of forming ant-tub boundary. (D-F) Sagittal sections of 11-somite chick after double fluorescent *in situ* hybridisation to detect *Shh*/*Foxg1*, shown in double (D) or single (E,F) channel view (*n*=3). Arrowheads indicate the *Shh-Foxg1* border. (G-I) Ventral views of flat-mounted isolated prosencephalon at HH10+ (11 somites, G) (*n*=2), HH12 (H) (*n*=3) and HH13 (I) (*n*=7), hybridised for *p57^Kip2^*/*Shh*. *p57^Kip2^* expands over HH10+ to HH13. Dotted lines indicate approximate ant-tub boundary. (J-L) At HH13, *p57^Kip2^* is detected anterior to *Shh* (blue line shows border; *n*=5). Scale bars: 100 µm in A,B,G-L; 50 µm in C-F.

As the hypothalamus develops, *Shh* undergoes dynamic change, becoming downregulated in the presumptive tuberal hypothalamus and upregulated in the anterior hypothalamus (Manning et al., 2006). To address whether this change occurs as *Fgf10^+^* progenitors develop into anterior progenitors, we examined *Shh* over 9-11 somites. At 9 somites, *Shh* expression in the prosencephalon (Fig. 6A) is similar to that of *Fgf10* (Fig. 3B), suggesting a brief period of co-expression. However, from 11 somites, *Shh* becomes downregulated in *Fgf10^+^* progenitors (Fig. 6B,C; Manning et al., 2006) and strong expression is now detected in cells at the periphery, including emerging anterior progenitors (Fig. 6B,C). Double *in situ* hybridisation and analysis of sagittal sections at 11 somites (HH10+) confirms that *Shh^+^* anterior progenitors abut the *Foxg1*^+^ telencephalon (Fig. 6D-F, arrowhead). Analysis of phospho-H3 (a marker of M-phase) and of *p57^Kip2^* suggests that at 11 somites, emerging anterior progenitors are already committing to a differentiation pathway: they upregulate *p57^Kip2^* (Fig. 6G; expression of *p57^Kip2^* is not detected in this region at 9-10 somites, data not shown) and show reduced phospho-H3 labelling, relative to neighbouring *Fgf10^+^* progenitors (Fig. 3H). *Shh^+^p57^Kip2^*^+^ cells rapidly increase in number along the antero-posterior axis (Fig. 6H,I)*.* Notably, however, at HH13, *p57^Kip2^* expression is detected anterior to *Shh* (Fig. 6J-L). This, together with our cell cycle and fate-mapping analyses, suggests that emerging anterior progenitors downregulate *Fgf10* and upregulate *Shh* and *p57^Kip2^*, then downregulate *Shh* as they differentiate.

### Shh signalling over HH10-13 is required for establishment of the neuroendocrine axis

We next investigated the mechanism that governs the growth and differentiation of anterior progenitors. Conditional manipulation studies in mouse and zebrafish have suggested a role for neuroepithelial-derived Shh in these events (Shimogori et al., 2010; Blaess et al., 2015; Zhao et al., 2012; Muthu et al., 2016; Orquera et al., 2016), but no study has examined whether this reflects a role for Shh in the early differentiation of *Fgf10^+^* progenitors.

To address this, we exposed HH9 (7-8 somite) embryos to cyclopamine, a pharmacological inhibitor of Shh signalling (Chen et al., 2002), then analysed embryos at progressive stages. Cyclopamine effectively (but transiently) inhibited Shh signalling, as judged by the downregulation of *Ptch1* in anterior progenitors at HH10-13 (Fig. S10; *Ptch1* is detected again by HH15). An additional consequence of inhibition of Shh signalling was the loss of detectable *p57^Kip2^* (Fig. S11).

**Fig. 7.**
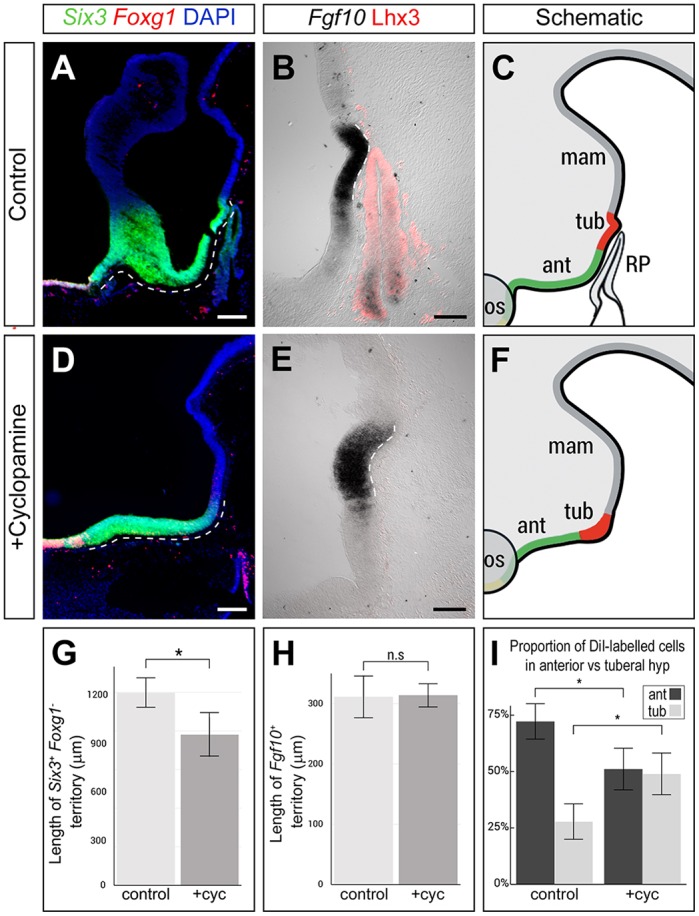
**Shh signalling at HH10-HH15 is required for differentiation of the anterior hypothalamus and RP.** (A-H) Analysis of *Six3/Foxg1* and *Fgf10*/Lhx3 on E5 sagittal sections following PBS (control) or cyclopamine treatment at HH9. (A,D,G) *Six3^+^Foxg1^−^* territory (dashed lines in A,D) is significantly shorter in cyclopamine-treated embryos than in controls (*P<0.05; two-tailed unpaired *t*-test; *n*=5 each). (B,E,H) The length of the *Fgf10^+^* tuberal territory (dashed lines in B,E) is not significantly affected in cyclopamine-treated embryos (*P*>0.05; two-tailed unpaired *t*-test; *n*=5 each). After cyclopamine exposure, the *Fgf10^+^* tuberal progenitor domain is significantly wider and broader (total volume 3.2±0.37×10^6^ µm^3^ compared with 1.45±0.05×10^6^ µm^3^ in controls; *P*=0.0032; *n*=4 embryos). In addition, the infundibulum is absent and RP fails to develop (rudimentary pouch in this embryo shown in Fig. S13D). Sagittal views in A,B are serial adjacent; sagittal view in E is 45 µm from that in D; schematics in C,F summarise data from sections between A and B, D and E, respectively. (I) A significantly higher proportion of DiI-labelled cells are retained in *Fgf10^+^* tuberal progenitors in cyclopamine-treated embryos compared with controls (*P<0.05; two-tailed unpaired *t*-test; *n*=4 each). Scale bars: 200 µm in A,D; 100 µm in B,E.

We then analysed anterior and tuberal progenitor domains at later times. All embryos in which Shh signalling was transiently eliminated showed consistent and predictable disruption to the anterior progenitor domain at E5. Although there was no significant difference in the length of the *Fgf10^+^* progenitor domain, the length of the *Six3^+^Foxg1^−^* progenitor domain was significantly reduced (Fig. 7A-H). *Six3* and *Fgf10* maintained their common caudal boundary in cyclopamine-treated embryos (Fig. S12). Together, this means that transient blockade of Shh signalling leads to the specific loss of anterior progenitor territory.

Notably, although the *Fgf10^+^* progenitor domain was not expanded in length in cyclopamine-treated embryos, it appeared significantly wider and broader than in controls (Fig. 7E,F). This raises the possibility that although blockade of Shh signalling prevents growth and differentiation of anterior progenitors, it does not prevent continued proliferation of *Fgf10^+^* progenitors, which then accumulate aberrantly. To test this, embryos were treated with cyclopamine or PBS at HH9, injected with DiI into region A at 9-10 somites, incubated until HH20, and then assayed for DiI labelling and *Fgf10* expression to obtain a quantitative picture of the contribution of cells to anterior versus tuberal progenitor domains. In control embryos, significantly more cells contributed to the *Fgf10^−^* anterior than to the *Fgf10^+^* tuberal progenitor domain. By contrast, in cyclopamine-treated embryos, there was a significant reduction to the contribution of cells to the anterior progenitor domain, and a significant increase to the contribution of cells to the *Fgf10^+^* tuberal progenitor domain (Fig. 7I). Thus, a larger proportion of DiI-labelled cells remain in the *Fgf10^+^* tuberal hypothalamus in cyclopamine-treated embryos. Together, this suggests a role for Shh signalling in the appropriate growth of anterior and tuberal hypothalamic domains.

**Fig. 8.**
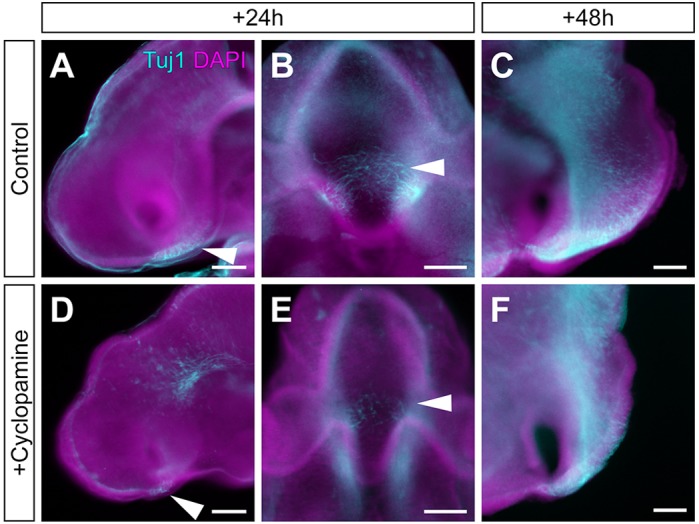
**Reduction in tract of the post-optic commissure (TPOC) after transient blockade of Shh signalling.** (A-F) TUJI immunolabelling of HH17 (A,B,D,E) or HH20 (C,F) embryos, in whole-mount lateral view (A,D), whole-mount ventral view (B,E) or isolated neuroepithelial view (C,F) after exposure to PBS (A-C, control) or cyclopamine (D,E). The TPOC (arrowheads) develops robustly in control embryos (A-C), but is significantly reduced in cyclopamine-exposed embryos after 24 h (88±52 µm, *n*=10) compared with controls (202±47 µm, *n*=5; *P*<0.01 two-tailed unpaired *t*-test) and 48 h (357±97 µm, *n*=8) compared with controls (623±78 µm, *n*=6; *P*<0.001; two-tailed unpaired *t*-test). Concomitantly, the anterior progenitor domain is reduced in length. Scale bars: 100 µm.

To determine the consequences of the loss of anterior progenitor territory on neuronal differentiation, we examined the tract of the post-optic commissure (TPOC), a tract that develops from the earliest-differentiating neurons in the anterior hypothalamus (Ware and Schubert, 2011). We detected a significant reduction in the number of neurons in the TPOC, both 24 h and 48 h after the transient blockade of Shh signalling (Fig. 8). Finally, in cyclopamine-treated embryos, the infundibulum was completely absent (*n*=8/10; Fig. 7D,E) or present only as a rudimentary evagination (*n*=2/10; Fig. S13). Additionally, these embryos displayed a spectrum of disruptions to RP. In each, a rudimentary pouch formed that expressed *Six3* (*n*=5/6) but did not express Lhx3 (*n*=4/4) (Fig. S13). In 7/10 embryos, the rudimentary pouch was not in close proximity to the ventral hypothalamus; instead, mesenchymal cells filled the intervening space (Fig. S13). Furthermore, the subset of cyclopamine-treated embryos that showed a rudimentary infundibulum exhibited a second ectopic rudimentary pouch-like structure (*n*=2/10; Fig. S13). Together, our studies reveal the importance of Shh signalling to the integrated development of the hypothalamo-pituitary axis.

## DISCUSSION

Here, we describe how rostro-caudal domains of the hypothalamus form in the embryonic chick. We show that the presumptive hypothalamus originates in neural tissue of the RDVM that lies directly above the PM and is composed of *Fgf10^+^* progenitors. These give rise to anterior (*Shh^+^Fgf10*^−^) and mammillary (*Emx2^+^Fgf10^−^*) progenitors through bi-directional (rostral and caudal) expansion, whereas a stable population of *Fgf10^+^* cells becomes restricted to the tuberal hypothalamus, including the infundibulum. Shh signalling is essential for the growth and differentiation of the anterior hypothalamus, including its resident neurons, and for development of the infundibulum and RP.

### *Fgf10* expression defines hypothalamic progenitors above the PM

Our fate-mapping studies define the location of hypothalamic progenitors at 9-10 somites. We pinpoint the position of anterior-most progenitors that will populate regions close to the optic stalk, posterior-most progenitors that will populate the caudal mammillary pouch and, along the medio-lateral axis, show that hypothalamic progenitors form a triangular territory that tapers posteriorly. This territory correlates with the expression profile of *Fgf10*, suggesting that *Fgf10* is a defining marker for hypothalamic progenitors. In support of this, at 9-10 somites, *Fgf10^+^* progenitors anteriorly abut *Foxg1* progenitors to define the hypothalamic-telencephalic interface.

Our data lend weight to the idea that the hypothalamus arises from part of the neural tube characterised by proliferating progenitors (termed the acroterminal region; Puelles and Rubenstein, 2015), but show conclusively that, at 9-10 somites, the prospective hypothalamus is underlain by the PM. In fact, the position and shape of the PM anticipates the developing hypothalamus and thus could facilitate signalling between the two tissues as late as 9-10 somites. Our previous studies have shown that BMPs become active in the PM at the 7-somite stage (Ellis et al., 2015), and have shown that BMP signalling from the PM is required for *Shh* downregulation and *Fgf10* upregulation in RDVM cells (Manning et al., 2006). These observations, and those here, suggest that BMP signalling from the PM induces *Fgf10* in overlying neuroepithelial cells, to define the presumptive hypothalamus.

### Development of anterior, tuberal and mammillary progenitor domains

We present numerous lines of evidence to show that *Fgf10^+^* progenitors give rise to *Fgf10^−^* anterior and *Fgf10^−^* mammillary progenitors. First, fate-mapping shows that *Fgf10^+^* progenitors contribute to the entire anterior and mammillary hypothalamus. Second, both phosphoH3 and EdU-labelling analyses reveal higher levels of proliferation in *Fgf10^+^* progenitors than in emerging *Shh^+^* anterior or *Emx2^+^* mammillary progenitors, suggestive of rostral and caudal growth fronts. Third, although *Fgf10^+^* progenitors proliferate rapidly, possibly in response to autocrine FGF signalling, the length of the *Fgf10^+^* territory remains remarkably constant over E1.5 (HH10) to E5 (the latest stage examined), whereas anterior and mammillary domains become apparent and then lengthen throughout this period. This suggests that the rate at which cells are retained by, and displaced from, the *Fgf10^+^* territory remains constant and ensures a stable population(s) of undifferentiated hypothalamic progenitors. We propose that displacement of cells away from the *Fgf10^+^* territory marks an early step in the differentiation of cells to an anterior or mammillary fate. In this process, cells become refractory to paracrine FGF signals from the progenitor domain, which could promote loss of the undifferentiated hypothalamic progenitor state.

As yet, we have not explored the factors that determine whether *Fgf10^+^* progenitors give rise to anterior versus mammillary progenitors. However, our studies suggest the position of *Fgf10^+^* progenitors along the A-P axis at 9-10 somites may restrict their fate: region A progenitors give rise to anterior and rostro-tuberal, but not mammillary, progenitors, whereas region C progenitors give rise to caudo-tuberal and mammillary, but not anterior progenitors. This could reflect an exposure to different extrinsic factors: anterior hypothalamic progenitors lie above the pharyngeal endoderm/prechordal mesoderm, and in close proximity to the oral ectoderm/hypophyseal placode/nascent telencephalon, whereas posterior hypothalamic progenitors lie above the prechordal mesoderm and close to the notochord/nascent midbrain. Further studies are required to investigate this.

### Shh signalling promotes the differentiation of *Fgf10^+^* to *Fgf10^−^* anterior progenitors

**Fig. 9.**
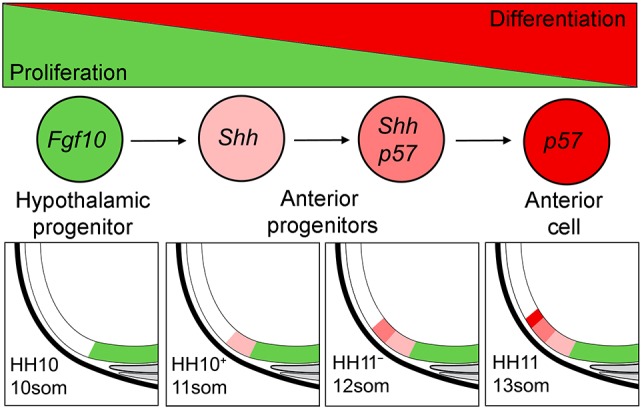
**Model for anterior hypothalamic development from *Fgf10^+^* progenitors.** The hypothalamus forms from a stable population of highly proliferative *Fgf10^+^* progenitors (green) that later contribute to tuberal domains (green). Shh signalling promotes the equal retention of *Fgf10^+^* progenitors and displacement of *Fgf10^−^* progenitors (light pink). Over time, such progenitors become displaced more rostrally and express *p57^Kip2^* (dark pink). These downregulate Shh and differentiate (red). Arrows indicate displacement of cells in caudal to rostral direction.

Here, we have focused on the mechanism that promotes anterior development and describe an essential role for Shh signalling (Fig. 9). We demonstrate, using cyclopamine treatment, that Shh signalling is required for *Fgf10^+^* progenitors to give rise to anterior progenitors. This, together with our cell cycle analyses, suggests that Shh acts on *Fgf10^+^* progenitors to promote their differentiation (Fig. 9). In this model, Shh is downregulated in *Fgf10^+^* progenitors but upregulated in emerging anterior progenitors (this study; Aglyamova and Agarwala, 2007), and Shh is then required to upregulate the differentiation marker *p57^Kip2^*. Such a mechanism suggests parallels to anterior hypothalamic development in zebrafish, where Shh signalling induces the transcription factor *rx3* to select *Shh^+^* anterior progenitors, then downregulates *rx3*, which allows progenitors to grow and differentiate appropriately (Muthu et al., 2016). Indeed, the proposed mechanism of action of Shh in growth and specification may be widely conserved: in mouse, conditional ablation of Shh from anterior hypothalamic cells prevents anterior hypothalamic differentiation (Shimogori et al., 2010; Zhao et al., 2012; reviewed by Blaess et al., 2015) and deletion of *Shh* from *Hesx1^+^* cells leads to a phenotype that closely resembles the one we describe here, including the loss of anterior hypothalamic cells, and disruption to the tuberal hypothalamus (Carreno et al., 2017).

Our work shows that the decrease in anterior progenitors after cyclopamine exposure leads to a reduction in differentiated neurons of the TPOC. We have not investigated whether specific subsets of neurons are absent, but conditional deletion of Shh in zebrafish and mouse leads to a loss of both ‘anterior’ and ‘tuberal’ neurons (Shimogori et al., 2010; Zhao et al., 2012; Muthu et al., 2016; Orquera et al., 2016; Carreno et al., 2017). Thus, we suggest that anterior *Shh^+^* progenitors will give rise to both differentiated anterior and tuberal neurons, a model supported through lineage-tracing studies in mouse (Alvarez-Bolado et al., 2012). Our studies are consistent with a model in which the earliest *Fgf10^+^* progenitors to express *Shh* and *p57^Kip2^* give rise to rostral-most hypothalamic neurons (e.g. those of the suprachiasmatic nucleus), then later ones differentiate to give rise to anterior neurons, and then the final ones give rise to tuberal neurons. In support of this, birthdating studies in mouse show that anterior neurons are generated prior to tuberal neurons (Shimada and Nakamura, 1973).

### Coordinated development of hypothalamo-pituitary neuraxis

What is the fate of *Fgf10^+^* progenitors that persist in the tuberal hypothalamus? Previous studies have shown that *Fgf10* persists in non-proliferating infundibular cells and proliferating collar cells that are retained at the ventricular zone (Pearson et al., 2011). We detect *p57^Kip2^*, a regulator of cell-cycle exit, in a subset of *Fgf10^+^* progenitors at HH18-26, and predict these are emerging infundibular cells. These require FGF signalling to form (Pearson et al., 2011), suggesting that infundibular cells can develop through prolonged exposure to autocrine FGF signalling. Collar cells, by contrast, do not immediately upregulate *p57^Kip2^* (Fig. S9), suggesting that they persist as proliferating cells or as cells that may enter quiescence at a later stage. Studies in chick and mouse have described *Fgf3/10^+^* stem-like cells within the tuberal hypothalamus that persist as quiescent cells in postnatal life (Hajihosseini et al., 2008; Pearson et al., 2011; Robins et al., 2013). Our studies raise the possibility that some descendants of embryonic *Fgf10^+^* progenitors persist as quiescent stem-like cells in the postnatal hypothalamus.

Our data reveal that Shh signalling is necessary for both development of the infundibulum and of RP: transient blockade of Shh signalling prevents formation of the infundibulum and prevents the differentiation of the Lhx3^+^ RP. A rudimentary pouch is detected, but is separated from the hypothalamus by a loose meshwork of mesenchymal cells. Parallel studies in mouse show a highly similar phenotype after conditional deletion of *Shh* from *Hesx1^+^* cells (Carreno et al., 2017). As yet, the mechanisms behind these phenotypes are not clear. Shh could act directly on each tissue – for example, in the tuberal hypothalamus, Shh could govern the transition of cycling *Fgf10^+^* progenitors to non-cycling infundibular cells (Pearson et al., 2011). Alternatively, the effects of Shh could be indirect: blockade of Shh signalling leads to abnormal accumulation of *Fgf10^+^* progenitors and abnormal broadening of the tuberal domain, which could in turn restrict infundibular development. Likewise, the failure of the infundibulum to form could, in turn, affect RP development (or vice-versa): many previous studies have suggested that reciprocal signalling events govern development of the infundibulum and RP, and that intimate contact between the two forming tissues is essential for their differentiation (reviewed by Davis et al., 2013; Rizzoti, 2015).

Nonetheless, severe disruptions to the neuronal hypothalamus, the infundibulum and RP reveal the importance of Shh signalling for the integrated development of the hypothalamo-pituitary neuraxis. Our work, together with that of Carreno et al. (2017), suggests that the action of Shh is evolutionarily conserved. Future studies using the chick embryo, an animal that is particularly well-suited to conditional interference, will allow us to determine whether Shh acts directly or indirectly to orchestrate development of the hypothalamo-pituitary axis and will enable us to probe the mechanisms that may direct *Fgf10^+^* progenitors to populate the anterior, infundibular or mammillary domains.

## MATERIALS AND METHODS

### Chick embryos

Fertilised Bovan brown chicken eggs (Henry Stewart & Co., Norfolk, UK) were staged according to Hamburger and Hamilton (1951). All experiments conformed to relevant regulatory and ethical standards (University of Sheffield). Hemi-dissection of E3-E5 chick embryos was performed by removal of retina, mesenchyme and ectoderm surrounding one half of the embryo. Neuroepithelial tissue was isolated after dispase treatment (Pearson et al., 2011).

### Fate mapping the ventral prosencephalon

Embryos (9-10 somites) were windowed and a dorsal incision made to access the ventral prosencephalon. Carbocyanine dyes, DiI (CM-DiI, Life Technologies) and DiO (Vybrant DiO, Life Technologies) were injected using a picospritzer II microinjection system (Parker Instrumentation). Injection sites were identified relative to the underlying PM and visualised through injection of blue food colouring (Dr Oetker).

### Cell proliferation

HH15 or HH20 embryos were treated *in ovo* with 200 µl of 0.5 mM 5-ethynyl-2′-deoxyuridine (EdU, Life Technologies) for 90 min, fixed with 4% PFA and sagittally cryosectioned. Proliferating cells were detected using the Click-iT EdU kit (Life Technologies) as described by the supplier's protocol. To quantify EdU-labelling density, the antero-tuberal or tubero-mammillary hypothalamus were each partitioned into eight equal lengths and the integrated density of each measured in Adobe Photoshop.

### Cyclopamine treatment

HH9 embryos were treated with 5 µl of 0.48 mM cyclopamine (Sigma) in PBS, pipetted over an incision in the vitelline membrane above the prosencephalon. After development, embryos were fixed in 4% paraformaldehyde, then processed by *in situ* hybridisation or immunohistochemistry. The optimal concentration of cyclopamine was determined through a concentration curve analysis.

### *In situ* hybridisation and immunohistochemistry

Whole-mount and cryosectioned embryos were analysed by immunohistochemistry and *in situ* hybridisation according to standard techniques (Manning et al., 2006). Fluorescent *in situ* hybridisation was performed using the TSA Plus Cyanine 3/Fluorescein System (PerkinElmer, NEL753001KT) according to the supplier's protocol. Primary antibodies used were: anti-LHX3 (1:50; 67.4E12, DSHB) and anti-TUJ1 (1:1000; Covance). Secondary antibodies used were Alexa Fluor 488 and 594 (1:500; Molecular Probes).

### Measurements of mRNA expression and morphological features

From HH10 to HH27, the tuberal progenitor domain was determined by measuring the length of *Fgf10* expression domain; the anterior progenitor domain was determined by subtraction of this from the *Six3^+^Foxg1^−^* antero-tuberal domain, measured in serial adjacent sections; the mammillary progenitor domain was determined by measuring the *Emx2^+^* territory. Measurements were carried out using ImageJ (v1.51).

### Statistics

Statistics were performed and graphs were generated in Microsoft Excel.
